# Striving for inclusivity: the crucial function of neurorehabilitation in the management of KIF1A syndrome

**DOI:** 10.3389/fneur.2024.1392858

**Published:** 2024-05-23

**Authors:** Seema Saini, Neelam Hitesh Tejani, Amrutkuvar Rayjade

**Affiliations:** ^1^Musculoskeletal Physiotherapy Department, Dr. D.Y. Patil College of Physiotherapy, Dr. D.Y. Patil Vidyapeeth, Pune, Maharashtra, India; ^2^Pediatric Neuro-physiotherapy Department, Dr. D.Y. Patil College of Physiotherapy, D.Y. Patil Education Society, Kolhapur, Maharashtra, India; ^3^Orthopedic Manual Therapy Department, Dr. D.Y. Patil College of Physiotherapy, D.Y. Patil Education Society, Kolhapur, Maharashtra, India

**Keywords:** KIF1A gene, KIF1A, neurorehabilitation, pediatric rehabilitation, physical therapy (PT)

KIF1A, a kinesin-like protein located on chromosome 2q37.3 and is expressed mainly in the brain and spinal cord and is crucial for axonal transport of synaptic vesicles and other cellular cargo, is integral to various cellular processes including meiosis, mitosis, and neuronal function (1). Its structure comprises distinct domains facilitating motor activity, cargo binding, and dimerization. Activation of KIF1A occurs upon cargo binding, enabling transport along microtubules (1). Initially identified in 2011, these illnesses often went undiagnosed due to limited access to comprehensive genetic testing methods like full gene sequencing and exome sequencing (1), later on in the year 2022 the prevalence of KIF1A estimated as 3–10/100,000 (2). Recent findings reveal *de-novo* mutations linked to brain atrophy and progressive encephalopathy. Classification includes pure and complicated phenotypes, with the latter featuring axonal neuropathy and cerebellar atrophy (1). Overall, KIF1A mutations contribute to a range of neurodegenerative disorders with varying inheritance patterns and clinical presentations (1). KIF1A-associated neurological diseases (KAND) encompass a spectrum of neurological conditions stemming from alterations in the microtubule motor protein KIF1A due to mutations in the KIF1A gene (1). Both dominant and recessive inheritance patterns are observed in various KAND variations. Researchers have identified diverse mutations in the same proteins among patients, with many displaying unique variants, prompting further exploration and discovery in KIF1A-related disorders (KRD) (1). Motor deficits, including muscle weakness, stiffness, and issues with balance and coordination, are common in people with KIF1A syndrome. This rare condition predominantly affects brain neurons but can also cause effects on muscles, nerves, and vision in addition to neurological manifestations. Some people with KIF1A syndrome may also have cognitive impairment because of brain neuron involvement, in addition to the motor abnormalities that are frequently associated with the condition. To treat these cognitive issues and maximize patients' functional independence and quality of life, cognitive rehabilitation is essential (1). Notably, research indicates that metabolic disorders including diabetes mellitus are associated with increased KIF1A gene expression and immunoreactivity (1). Gene therapy is a promising treatment option for KAND, while specific proof of its effectiveness is still pending. The use of nanoparticles, engineered microRNA, plasmid transfection, viral vectors like adenovirus, herpes-simplex virus, lentivirus, and recombinant adeno-associated virus (rAAV), as well as technologies like polymer-mediated gene delivery and CRISPR-based therapeutics, are just a few of the experimental strategies that researchers are investigating (3). By focusing on the KAND-causing genes, these approaches aim to increase the lifespan and function of neuronal cells. There is still no approved standard drug or surgical intervention for KAND, despite these advancements. Gene-based therapies are therefore considered to be crucial for the treatment of this disorder (3, 4).

Physiotherapy can play a more significant role in the management of neurological illnesses linked to KIF1A than just treating physical symptoms. It takes into account the various difficulties that people with KIF1A syndrome may encounter, such as chronic pain, motor impairments which leads to restrictions on daily activities (5). Physiotherapy is an integrated strategy (6). Physiotherapists use a range of treatments, including stretching exercises, manual therapy, and focused exercises, to treat motor deficiencies such as stiffness, weakness, and problems with balance and coordination. In addition to increasing muscle strength, flexibility, and motor control, these interventions work to improve the mobility and gait patterns required for carrying out daily activities like sitting, standing, walking, and reaching (7). Furthermore, the neurological symptoms of KIF1A syndrome frequently cause chronic pain, discomfort, and muscle tightness in its affected persons (1). Manual therapy, myofacial release, and gentle stretching techniques are therapies that can aid with pain relief, reducing muscle tension, and improving overall comfort (8). Physiotherapists are essential in fostering functional independence and improving activities of daily living in addition to managing symptoms. For people with KIF1A syndrome, physiotherapist can suggest adapted equipment to help them become more independent in their daily lives. Physiotherapy can greatly improves the overall quality of life for people with KIF1A syndrome by addressing motor deficits, controlling pain, encouraging functional independence, and offering continuing support and education. Despite the difficulties presented by KIF1A-associated neurological illnesses, physiotherapists enable patients and their families to enhance physical function and participate more fully in daily activities through individualized interventions and an integrated approach to care.

While the focus of treatment for KIF1A often revolves around managing symptoms and improving quality of life, physiotherapy can play a crucial role in the multidisciplinary approach to care for individuals with KIF1A syndrome (9). Physiotherapy can play a key role in promoting functional independence and enhancing activities of daily living for individuals with KIF1A syndrome. Therapists focus on improving mobility, gait patterns, and motor skills necessary for tasks such as sitting, standing, walking, and reaching. Adaptive equipment and assistive devices may also be recommended to support independence and optimize function in daily activities (9). Many individuals with KIF1A syndrome experience chronic pain, discomfort, and muscle tightness as a result of their neurological symptoms. Physiotherapy interventions such as manual therapy, massage, and gentle stretching techniques can help alleviate pain, reduce muscle tension, and improve overall relief (9). Physical therapists work collaboratively with other healthcare professionals to develop comprehensive pain management strategies tailored to each individual's needs (10). By addressing motor impairments, promoting functional independence, and managing pain, physiotherapy can play a vital role in enhancing the overall quality of life for individuals with KIF1A syndrome. Through continuous support, education, and encouragement, therapists enable patients and their families to maximize physical function and engage more fully in everyday activities. Individuals with KIF1A syndrome can benefit from frequent physiotherapy treatments by improving their strength, mobility, and general wellbeing, which will enhance their quality of life. Physiotherapy can be extremely important in the multimodal management of KIF1A syndrome since it can help with pain management, functional independence promotion, motor deficits, and general quality of life improvement. Physical therapy accessibility can be impacted by a number of factors, including financial resources, geographic location, the availability of specialized facilities, and means of transportation. The degree to which caregivers or family members support and participate in a child's ability to attend and participate in in-person treatment sessions can have a significant effect. Community members' knowledge of KIF1A syndrome may have an impact on their understanding of and acceptance of physical therapy treatments for children with the condition. Social status, cultural beliefs, and the stigma attached to disabilities can all have an impact on how important physical care is prioritized in a family or society. Administrative processes, insurance coverage, waiting periods, and coordination between several healthcare providers may make it challenging to receive timely and comprehensive physical therapy services. To address these issues, a multidisciplinary approach comprising collaboration between healthcare professionals, educators, legislators, community organizations, and families is required. By being aware of and responding to these constraints, healthcare practitioners can assist children with KIF1A syndrome receive and benefit from physical therapy services more effectively. To effectively address the special needs of people with KIF1A syndrome, physiotherapists need to be more aware of the condition and receive specialized training. KIF1A syndrome is a genetic illness that needs constant attention and assistance and it can degrade the quality of life of the child. Through personalized interventions and ongoing support, physiotherapists can help individuals with KIF1A syndrome maximize their physical potential and achieve greater independence and wellbeing. Interventions in physical therapy must be long-lasting and flexible enough to adjust as a patient's condition does. In order to evaluate the success of physiotherapy interventions and make the required modifications to the treatment plan, long-term monitoring and follow-up are needed and so further it can lead to improved quality of life. Addressing these challenges will require concerted efforts from healthcare professionals.

## Author contributions

SS: Formal analysis, Investigation, Methodology, Validation, Writing – review & editing. NT: Conceptualization, Methodology, Software, Writing – original draft. AR: Funding acquisition, Resources, Validation, Writing – review & editing.
